# Back to the Future for Influenza Preimmunity—Looking Back at Influenza Virus History to Infer the Outcome of Future Infections

**DOI:** 10.3390/v11020122

**Published:** 2019-01-30

**Authors:** Magen Ellen Francis, Morgan Leslie King, Alyson Ann Kelvin

**Affiliations:** 1 Department of Microbiology and Immunology, Faculty of Medicine, Dalhousie University, Halifax, NS B3K 6R8, Canada; M.Francis@dal.ca (M.E.F.); MorganKing@dal.ca (M.L.K.); 2 Department of Pediatrics, Division of Infectious Disease, Faculty of Medicine, Dalhousie University, Halifax, NS B3K 6R8, Canada; 3 Canadian Centre for Vaccinology, IWK Health Centre, Halifax NS B3K 6R8, Canada

**Keywords:** immune response, original antigenic sin, imprinting, preimmunity, influenza virus, antigenic drift, antigenic shift, orthomyxoviridae, Spanish influenza, pandemic

## Abstract

The influenza virus-host interaction is a classic arms race. The recurrent and evolving nature of the influenza virus family allows a single host to be infected several times. Locked in co-evolution, recurrent influenza virus infection elicits continual refinement of the host immune system. Here we give historical context of circulating influenza viruses to understand how the individual immune history is mirrored by the history of influenza virus circulation. *Original Antigenic Sin* was first proposed as the negative influence of the host’s first influenza virus infection on the next and *Imprinting* modernizes *Antigenic Sin* incorporating both positive and negative outcomes. Building on imprinting, we refer to *preimmunity* as the continual refinement of the host immune system with each influenza virus infection. We discuss imprinting and the interplay of influenza virus homology, vaccination, and host age establishing preimmunity. We outline host signatures and outcomes of tandem infection according to the sequence of virus and classify these relationships as monosubtypic homologous, monosubtypic heterologous, heterosubtypic, or heterotypic sequential infections. Finally, the preimmunity knowledge gaps are highlighted for future investigation. Understanding the effects of antigenic variable recurrent influenza virus infection on immune refinement will advance vaccination strategies, as well as pandemic preparedness.

## 1. Introduction: Influenza Viruses are the Archetype Emerging and Re-Emerging Viruses

Influenza virus is not a single virus but, instead, a family of constantly changing virus strains. The family is defined by four virus types that are then divided into subtypes or lineages. Influenza viruses are the archetype of emerging and re-emerging viruses. Influenza virus type A and type B currently circulate in humans causing seasonal epidemics and occasional pandemics [1,2,3]. Due to the propensity for mutation, distinct strains emerge each year with the ability to infect the vulnerable human population [4]. Yearly, influenza viruses infect between 5% and 15% of the population with higher incidences (~30%) in children [5]. This suggests that every person will be infected or exposed to the influenza virus by the time they are four years old. Furthermore, considering prominent government-supported influenza vaccination programs, the age of first influenza virus exposure is most likely lower. Since influenza viruses cycle yearly in the human population and the immunity gained from previous infection is not life-long [6], people are exposed to influenza viruses recurrently. This means that everyone, with the exception of newborn infants, has an influenza immune history.

Humans build a complex immune history due to repeated exposures with new influenza strains from infection and seasonal vaccinations. The influence of preimmunity was first proposed in the 1960s. Dr. Thomas Francis Jr. suggested that a person’s first influenza virus infection would be deleterious to the clinical outcome of the next infection and referred to this as *Original Antigenic Sin (OAS)* [7]. The mechanisms of this outcome were regulated by memory immune responses. The re-stimulated memory immune responses directed toward epitopes of the first virus were thought to cause enhanced disease by failing to control the second viral infection [7]. Subsequently, the OAS phenomenon is now referred by other terms such as *Antigenic Seniority*, *Negative Interference, Back-boost,* or *Negative Antigenic* Interaction [7,8,9,10,11].

**Figure 1:**
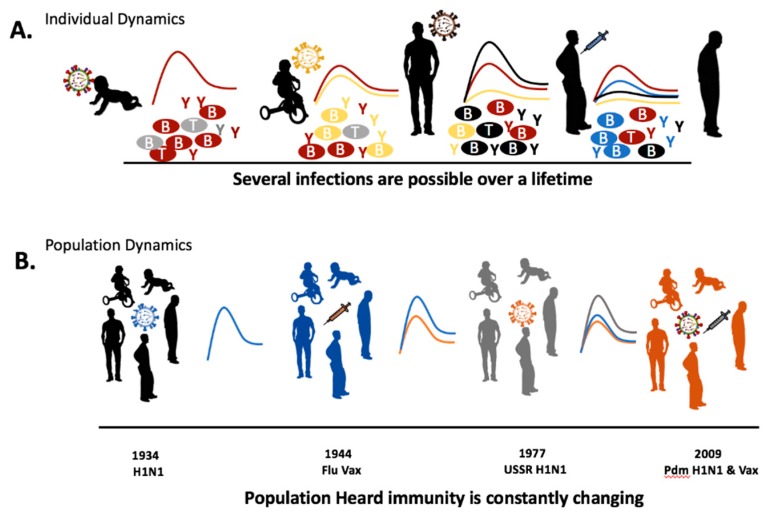
Influenza immune background development following recurrent infections and vaccinations. The schematic illustrates possible immune fluctuations following influenza virus infection or vaccination. Immune fluctuations and potential protection can be conceptualized at both the individual or population level. The heterogeneity of the immune background is dependent on the sequence of virus exposures and their genetic and antigenic relatedness. Immune specificity that is gained during influenza virus immune imprinting during infancy or childhood leading to the generation of virus-specific T and B cells are retained at some level throughout life. T = T cell; B = B cells; Y = antibodies.

We know that the interaction between the immune system and an evolving pathogen is more complex than embodied by OAS. Immune history is not simply composed of the host’s first influenza virus infection. Rather, the interaction between the immune system and influenza viruses is dynamic with each exposure. Each host-pathogen interaction shaping the individual immune landscape and, by extension, the immune landscape of the community (Figure 1). Without detailing the immune mechanism, previous studies have indicated that the order of virus infections and the viruses’ antigenic relatedness between the viruses has significant impact on the adaptive immune cell population or antibody pools [12,13,14]. For example, secondary infection with a virus of the same lineage has been hypothesized to invoke antibodies directed toward the hemagglutinin HA head while a secondary infection of a different viral lineage will elicit antibodies targeting conserved viral regions, such as the HA stalk [15]. The constant circulation of a heterogeneous pool of influenza viruses allows for many possible permutations for viral infection order. In this review, we will analyze the order of viral exposures depending on the homology of the viruses. Specifically, we refer to these as (1) monosubytypic homologous, (2) monosubtypic heterologous, (3) heterosubtypic, and (4) Heterotypic sequential infections (Table 1). The influence of a previous influenza virus exposure on the outcome of a subsequent infection may be both beneficial and deleterious. A person’s first influenza infection is a significant event, the imprinting event, which creates the largest background pool of long-lasting immunological memory cells [16]. Subsequent infections lead to cumulative effects of influenza viruses on the immune background which shape future responses [17]. We propose that a person’s immune history of influenza virus infections should be known as preimmunity. The schematic shows how an individual’s immune landscape may evolve during seasonal exposures to influenza viruses and vaccinations (Figure 1). The imprinting event that occurs during early life exposure leads to virus-specific immune B cell and T cell memory clones that are long-lived. Specifically, for the humoral arm of the immune system, imprinting leads to the development of antibodies that target surface and internal viral epitopes specific to this first influenza virus which remain prominent throughout life [18]. Most antibodies will target the immunologically-dominant HA molecule, but other viral antigenic epitopes may dominate in later infections [19]. The host immune composition varies with each infection in respect to antibody specificity and B cell longevity. Following a new influenza virus infection, the resulting immune constellation is dependent on the synergy between the pre-existing immune specificity and the characteristics of the next influenza virus infection (virus strain, subtype, lineage, or type).

Below we review the literature surrounding influenza virus immune imprinting and preimmunity. We first outline the development of preimmunity by discussing influenza virology, influenza history, and primary immune responses. This sets the stage for integrating modern investigations of imprinting and preimmunity. We conclude by highlighting the mechanistic knowledge gaps. Advancing the field of host preimmunity will contribute to the next generation of influenza vaccines.

## 2. Influenza Virus Biology Enables Frequent Antigenic Change

**Figure 2:**
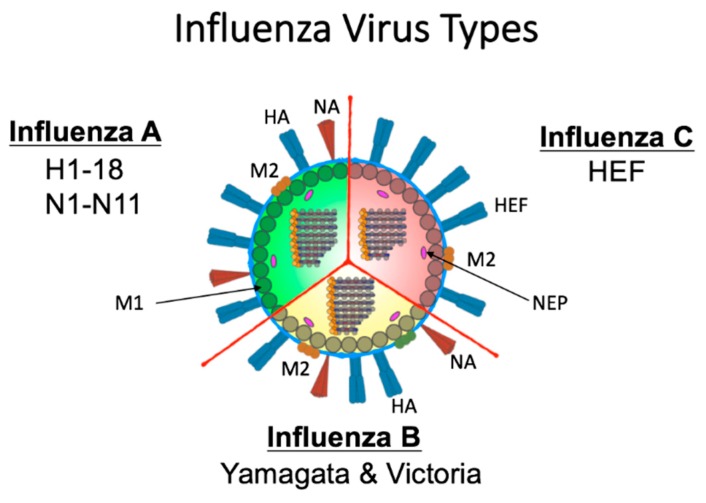
Schematic of Influenza A, B, and C virus structure. Influenza A is defined by its surface proteins hemagglutinin (HA) and neuraminidase (NA) of which there are 18 HA and 11 NA. Influenza B viruses are categorized into two lineages (B/Yamagata and B/Victoria). The surface of both A and B viruses contains HA, NA, and the M2 proteins. Internally, A and B viruses both have eight genomic segments. Influenza C viruses have only one external spike protein (HEF) which functions both in viral entry and egress and an ion channel M2 protein. Type C viruses have 7 internal genomic segments. M1 protein line the envelope internally adjacent nuclear export protein (NEP) proteins for type A, B, and C viruses. The genomic segments are encapsidated by the NP protein and form the ribonucleocapsid with the polymerase proteins Polymerase acidic (PA), Polymerase basic 1 (PB1), and Polymerase basic 2 (PB2).

The segmented RNA genome of the influenza virus family (Orthomyxoviridae) facilitates antigenic variability [20]. The family is categorized into four antigenically distinct virus types: A, B, C (Figure 2), and D [21,22]. Briefly, influenza C and D viruses are not regarded as health threats. In humans, influenza C virus causes mild illness while type D virus is incapable of human infection [23]. The surface of influenza C virus is defined by a single spike protein referred to as hemagglutinin-esterase-fusion glycoprotein (HEF) [23] (Figure 2). This protein has host receptor binding abilities, membrane fusion capabilities, and enzymatic activity for egress [23]. Although antibodies to influenza C virus are detected early in life [23], little is known about how infection contributes to host immune background. Herein, we will concentrate on A and B virus types which cause significant disease outbreaks [24].

Both type A and B consist of eight negative-sense, single-stranded RNA segments: Polymerase acidic (PA/PA-X), Polymerase basic 1 (PB1/PB1-F2), Polymerase basic 2 (PB2), HA, nucleoprotein (NP), NA, matrix protein 1 and 2 (M1/M2), non structural protein 1 (NS1), and nuclear export protein (NEP/NS2) [25]. PB2, PB1, and PA make up the RNA-dependent RNA polymerase complex which have been linked to disease severity [26]. Together, these proteins have roles in mRNA cap recognition, RNA elongation, endonuclease activity, and protease function [25,27]. NP, M1, M2, and NEP/NS2 proteins function in nuclear import, virus uncoating, and the nuclear export of RNA [25].

The HA and NA [25] surface proteins are the immunodominant proteins of the influenza virus which is why they have been the focus of many preimmunity studies [12,28,29,30,31,32]. Influenza A viruses are capable of moving between animal and human hosts, permitting a large degree of disease and destruction. For type A viruses there are 18 different hemagglutinin subtypes (H1 through H18) and 11 different neuraminidase subtypes (N1 through N11) [33,34] that combine to define the A virus subtype (e.g., H1N1). Extensive antigenic diversity exists within each subtype as each strain mutates during host infection. Influenza B virus has a more limited host range and has only been shown to infect humans, seals, and experimental animals such as ferrets [35,36]. Type B viruses are not categorized by subtypes but instead are defined by two lineages (B/Victoria lineage and B/Yamagata lineage), each of which is also comprised of antigenically evolving strains [37]. The NA of both type A and B viruses has sialidase enzymatic activity which functions during viral budding and egress [33].

Molecular analysis of HA showed that it is comprised of an immunodominant globular head domain distal to the viral envelope and a proximal membrane stalk domain [38]. The HA molecule binds sialic acids which can be in α2,3- or α2,6-linkage to galactose. Binding of HA to sialic acid-linked host proteins regulates virus cell entry [33]. The HA globular head is variable and frequently mutated. Since the host cell receptor binding region is located in the head domain, it is a target for antibodies enabling virus neutralization. The stalk domain is conserved among viral HA proteins and functions during host-virus membrane fusion leading to genome uncoating [39]. There is considerable homology in the HA stalk among type A viruses which are classified into two groups. Group 1 is comprised of H1, H2, H5, H6, H8, H9, H11, H12, H13, and H16 [33]. Group 2 includes H3, H4, H7, H10, H14, and H15 [33]. Antibodies targeting the stalk region have been shown to broadly block membrane fusion and viral infection across viruses per HA group [39].

Influenza viruses have two main modes of mutational change: antigenic shift and antigenic drift enabling repeat infection [6]. Antigenic drift is the accumulation of small mutations in the viral genome leading to antigenic changes over time [40]. Conversely, antigenic shift is an abrupt change in that results from the rearrangement of genomic RNA segments between two or more influenza viruses [41]. Antigenic Drift leads to seasonal influenza epidemics while shift is the cause of pandemic influenza virus outbreaks [41]. Both avenues lead to antigenically divergent viruses that may no longer be fully recognized by the immune system.

## 3. Host Immune Responses to Primary Influenza Virus Infection

Using experimental studies, the early stages of a host’s first influenza virus infection have been defined by the antiviral response which lends to the initiation of antigen-specific adaptive immunity [40]. By summarizing the knowledge of the immune responses to primary influenza virus infection we create a reference point for the dissection the immune programs of secondary and tertiary infections.

Nonspecific effectors are mobilized for blocking viral replication, containing virus dissemination, and recruiting effector cells for viral clearance [42]. Since influenza viruses are transmitted through the respiratory route, the initial site of infection for influenza viruses is the respiratory epithelium [40,43,44,45]. Specifically, ciliated pseudostratified columnar respiratory epithelial cells from the trachea, bronchi, nasal cavity, and submucosal glands, as well as type I and II pneumocytes of the alveoli are targeted. After infecting the epithelium, the virus spreads to leukocytes such as macrophages, dendritic cells (DCs), and Natural Killer (NK) cells [40]. Infected cells sense viral PAMPS (pathogen associated molecular patterns) through cell specific PPRs (pattern recognition receptors) [46]. There are three families of receptors that sense viral PAMPs: TLRs (toll-like receptors), RIG-I (retinoic acid inducible gene—I), and NOD-like (nucleotide oligomerization domain-like) receptors [40,47,48,49]. Stimulation of these receptors induces the expression IFN-α, chemokines, and other immune orchestrating proteins. These immune mediators induce an antiviral state by inhibiting viral spread and recruiting specific immune cells for viral clearance [40]. NK cells aid in influenza viral clearance by recognizing and lysing influenza virus-infected cells [46]. Alveolar macrophages phagocytose virus infected cells for antigen presentation. Both conventional and plasmacytoid DCs have a central role bridging innate immunity to the targeted adaptive immune responses during influenza virus infection [50]. Conventional DCs sit under the epithelium and lung parenchyma to continuously survey the airway environment with their dendrites [34]. Detection of influenza virus initiates DC up-regulation of the chemokine receptor CCR7 for migration to the draining lymph node [50]. In the lymph node, DCs present viral antigen to CD4^+^ T cells, CD8^+^ T cells, and B cells. CD4^+^ T cells differentiate into Th1 or Th2 cells depending on other exogenous signals (polarizing cytokines such as IL-2, IL-12, IL-4). CD8^+^ T cells up-regulate chemokine receptors (CXCR3 and CCR4) to home back to the lung to kill virus infected cells. Global gene expression analysis of influenza infection in ferrets has shown that the host immune responses are biphasic [51,52]. Early robust innate ISG (interferon stimulated genes) and chemokine response that were initially generated begin to shut down (day 7 post inoculation) as an adaptive immune phase becomes prominent. This “switch” is the elimination point of respiratory viral load and shutting down the inflammatory response, which contributes to lung pathogenesis [51,52].

Much of this review will concentrate on the humoral immune response during influenza virus infection in the preimmune host. With this in mind, we profile the role of CD8^+^ T cells in the host-pathogen interaction for comparison in the sequential infection studies that we discuss below. CD8^+^ T cells contribute to the clearance of virally infected cells by several different actions much of which is regulated by respiratory DC produced cytokines. As mentioned above, DCs (CD103^+^ DCs) that detect virus within the lungs, migrate to the lymph node transporting with them the acquired influenza virus antigen. In the lymph node, naïve CD8^+^ T cells interact with CD103^+^ DCs which leads to T cell activation, proliferation, and survival [53,54]. The effector CD8^+^ T cells migrate to the lung where they contribute to the immune response by releasing proinflammatory cytokines such as IFN-γ, TNF-α, MIP-1α to activate additional immune responses. In the lung, CD8^+^ cytotoxic T cells also directly kill infected cells by releasing perforin and granzyme or by activating proapoptotic TNF receptors in the surface of the target cell. These activities are directed by the cell type: epithelial cells (CD45^−^) or DCs (CD45^+^ CD11c(hi)) [55]. Further expansion in the lungs is initiated by CD28 co-stimulation by DCs which maintain CD8^+^ T cell responses promoting viral clearance [56]. Once in the lung, CD8^+^ T cells acquire the ability to produce the regulatory cytokine IL-10 inhibiting continual CD8^+^ T cell infiltration and the activity of Th17 cells [57,58]. Moreover, considering the role of CD8+ T cells during secondary infection, CD8^+^ T cells have greater potential for cross-reactivity between antigenically divergent influenza virus strains since these cells can target internal conserved viral proteins [54]. Although T cell cross-reactivity cannot inhibit viral infection, the presence of previously activated CD8^+^ T cell specific clones can increase the recovery time of influenza virus secondary infections.

The primary humoral response to infection is defined by the generation of short-lived plasma cells (antibody producing cells) that reside in secondary lymphoid organs [59]. Activated B cells mature into plasmablasts and plasma cells producing antibodies targeted at viral epitopes. During primary influenza virus infection, a large and diverse pool of antibodies are produced and circulated [17]. Animal modeling of influenza virus infection in naïve hosts has shown that most elicited antibodies are HA head-specific [31]. Smaller percentages of elicited antibodies target the HA stalk, NA, M2, or internal proteins of the virus [28]. Importantly, antibodies directed toward the conserved HA stalk can be broadly reactive and block membrane fusion (discussed above) [60,61]. Several antigenic sites and B cell epitopes (150) have been identified on the HA molecule [62]. These have been shown to be important for host protection by neutralizing the virus, inhibiting viral fusion, and promoting viral clearance [62,63,64].

B cells are expanded rapidly, then decline as immunological memory is established. In the germinal centre, B cell specificity is refined for improved responses toward the pathogen. Refinement occurs by somatic hypermutation (SHM), class-switch recombination (CSR), and clonal selection [65] which together are referred to as affinity maturation. Selected memory B cells are established for long-term circulation to protect against subsequent infections. They may be germinal center-dependent or germinal center-independent depending on how the cell developed [66]. Strain-specific memory B cells are lifelong since antibodies specific to historical influenza viruses have been detected in humans decades after initial infection [18].

The adaptive cellular and humoral responses move through 3 phases: expansion, contraction, and at last memory. Expansion is the time period when antigen specific T and B cells proliferate to produce a large number of reactive cells to control the pathogen. Experimental studies from our group as well as others investigating immune responses in animal models (non-human primate and ferret) have shown that lymphocytes as well as virus specific antibodies circulate at high numbers (in the expansion phase) even after pathogen clearance (21 and 48 days, respectively) [32,67]. Eventually, adaptive immune cells begin to contract (contraction phase) until there is a limited number of highly specific cells able to circulate for immune surveillance. This memory pool of pathogen specific cells establishes long-term protection for secondary encounters [68].

## 4. Recurrent Infection and Immunological Memory

Infection-reinfection or sequential infection experiments attempt to recapitulate influenza seasonality using animal models. The host is primed with an initial influenza virus before a challenge with a secondary infection following a recovery period. From infection-reinfection studies, it is clear that re-challenge with the same or a similar pathogen invokes memory activation of the humoral and cellular arms of the adaptive immune system [13,32,51,52]. Antibodies directed toward the HA molecule can block viral entry into cells if the host is re-exposed to the same virus strain [13]. Above we defined the exposure permutations in terms of influenza virus antigenicity as monosubtypic homologous, monosubtypic heterologous, heterosubtypic, and heterotypic reinfection relationships [32]. In immunocompetent individuals, sterilizing immunity is achieved via antibody-dependent virus neutralization during a homologous reinfection [69]. By contrast, heterologous reinfection, whether monosubtypic heterologous, heterosubtypic, or heterotypic is often non-sterilizing since viral antigenic sites has changed [13,32]. Non-sterilizing events involve cellular CD8^+^ responses that target viral internal proteins (as defined above) [54]. There is limited information regarding the involvement with other aspects of adaptive immunity such as memory NK cells during a heterologous reinfection event. Virus exposure following a primary infection most often occurs during the memory phase, not the expansion or contraction phase of the primary adaptive immune response. For the influenza virus-host interaction, the interval of time between a primary and secondary exposure is yearly due to virus seasonality and vaccination programs [70].

## 5. Epidemiological History of Influenza Virus Circulation Mirrors the Human Influenza Background

The history of influenza virus circulation is encompassed within the individual. In each person lies the timeline of past epidemics materialized as memory cell specificity. Since the immune responses of each new infection is dictated by individual preimmunity, we look to the past to predict the outcome of the next infection. Here we will briefly review the modern history (past ~150 years) of influenza virus circulation to understand the epidemiological and immunological outcomes of influenza virus infection and vaccination.

**Figure 3:**
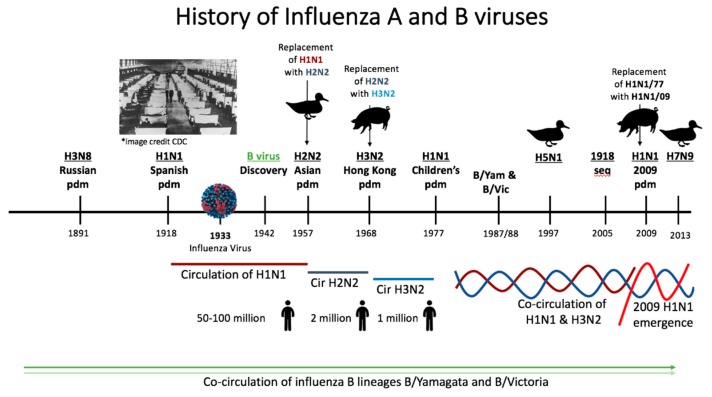
Timeline of the history of influenza virus circulation in humans since 1890s. The timeline shows the history of influenza virus type A and B circulation since 1890. There have been 6 presumptive pandemics in this time period. The Russian pandemic of ~1891 occurred prior to the discovery of the influenza virus and is known only by historical medical records. Seroarcheology has suggested that this outbreak was caused by an H3N8 virus. In 1918 what was known as the Spanish Influenza Pandemic took place. The H1N1 virus which had caused the pandemic was not definitively identified until lung samples from victims of the pandemic were sequenced in 2005. It is estimated that 50 to 100 million deaths were caused by this virus. H1N1 viruses circulated until 1957 when H1N1 was replaced with an H2N2 pandemic virus during the Asian pandemic. The H2N2 virus was replaced with H3N2 during the 1968 Hong Kong pandemic. In 1977, H1N1 resurfaced and since then, H1N1 and H3N2 have co-circulated. In 2009, the emergence of an H1N1 strain of swine origin replaced the former seasonal H1N1 virus ‘lineage’ which was in circulation from the 1977 lineage. In addition to circulating seasonal influenza viruses, avian virus spillover events also lead to human infection. Major avian virus spillover events occurred in 1997 and 2013 for the influenza A viruses H5N1 and H7N9, respectively. During this time period, influenza B viruses have also co-circulated in humans. *Image credit CDC for the 1918 pandemic and the influenza virion [79].

The influenza A virus was first identified as the etiological agent of human influenza in 1933 shortly after the advent of electron microscopy [71]. Influenza B virus was discovered in the following decade [72,73] (Figure 3). Prior to virus discovery, the agent responsible for influenza-like epidemics/pandemics such as the 1918 influenza pandemic were not readily determined [74]. The constructed timeline (Figure 3) starting in 1889 shows the history of influenza A and B virus circulation in the human population. There have been six presumptive pandemics caused by influenza A viruses since 1889 that have defined the immunological landscape of humans. The Russian pandemic of 1889 and the 1918 pandemic occurred prior to the discovery of the influenza virus [1,6]. The etiology of these disease outbreaks was determined by seroarcheological studies by sampling people who had lived prior to and during the time of the epidemics. Seroarcheology and molecular clock analysis suggested an H3N8 virus and an H1N1 virus as the etiological agent of the 1889 and 1918 pandemics, respectively [74,75]. It wasn’t until the 1918 virus was sequenced (1997) then resurrected in 2005 that H1N1 was definitively shown to be the causative agent of the 1918 pandemic [26,76]. It is estimated that the 1918 H1N1 virus caused 50 to 100 million deaths worldwide between 1918 and 1919, establishing it as the deadliest pandemic known to modern history. Sequencing has suggested that the virus has both avian and swine origins [26,27,76,77]. After 1918 there were several introductions of H1N1 viruses into human circulation from zoonotic sources. Therefore, the H1N1 viruses which circulated until 1957 were not direct descendants of the original 1918 H1N1 virus but instead originated from other spillover events [75]. In 1957, H1N1 was replaced with an H2N2 virus during the Asian pandemic. This outbreak was responsible for 2 million deaths [1]. The H2N2 virus was usurped by the introduction of the influenza A virus subtype H3N2 of swine origin which emerged during the Hong Kong pandemic of 1968 [74]. The driving factor causing H2N2 virus replacement with H3N2 subtype has been hypothesized to be the population’s previous immunity to sub-immunodominant domains on the influenza virus, i.e., the HA stalk and the neuraminidase [28]. H3N2 circulated as the only influenza A virus for nine years, until H1N1 resurfaced in 1977. A possible laboratory escape, the 1977 resurfacing of the H1N1 virus is referred to as the Children’s Pandemic since older individuals were protected due to preimmunity to H1N1 viruses [1,78]. Since 1977, H1N1 and H3N2 have co-circulated in humans.

In 2009, the emergence of an antigenically novel H1N1 “lineage” caused the sixth influenza virus pandemic [80]. The virus, which was a reassortment of human, swine, and avian influenza viruses, replaced the former seasonal H1N1 virus “lineage” which had circulated since 1977 [80].

Aside from the human circulating A viruses, virus spillover events originating in animal species have become increasingly more common and their influence should also be considered as a force shaping human preimmunity and imprinting [81]. Human infections of zoonotic strains of other influenza A virus subtypes such as H5N1, H6N2, H7N1, H7N7, and H9N2 have been reported [81,82,83,84,85,86]. Human infections of avian H5N1 was first detected in 1997 [82]. In 2013 H7N9 became a significant problem to human health in China where frequent spillovers have caused hundreds of human infections [81,83].

During 1891 to present day, influenza B viruses have been co-circulating, creating a complex immune landscape [73]. Influenza B was identified in 1942 and added to the influenza vaccine to create a bivalent vaccine [73] (Figure 3). The WHO initiated circulating influenza virus surveillance in 1973, which led to the discovery of the B viruses lineages B/Yamagata/16/1988 (B/Yamagata lineage) and B/Victoria/2/1987 (B/Victoria lineage) in the late 1980s [73].

By viewing the history of influenza virus circulation through the lens of imprinting, researchers can make assumptions about individual host imprinting and immune history. The influenza virus strain that an individual imprinted upon can be deduced by cross-referencing birth year with strain predominance [16]. For example, we can conclude that people born between 1918 and 1957 were imprinted with strains of H1N1 viruses since it was the only subtype of influenza A circulating in those years [16]. Those born between 1957 and 1968 would have imprinted against H2N2 and those born between 1968 and 1977 imprinted against H3N2 according to circulation history. For young individuals during H3N2 and H1N1 virus co-circulation, the predominant subtype per birth year is considered to be the imprinting virus. Since there was a sequential emergence and replacement of the influenza A virus subtypes (H1N1, H2N2, and H3N2) the current population, there is much variability for imprinted immunity. Moreover, the sequential nature of influenza virus circulation has led to heterosubtypic infection-reinfections throughout history. For instance, people born in 1947 imprinted with H1N1 and were reinfected with an H2N2 strain in ~1957. This sequence of infections can represent a person born in 1947 living through 1957 and would be noted as H1N1->H2N2. Additionally, the 2009 surfacing of an antigenically novel H1N1 strain (2009 H1N1) gave opportunity for monosubtypic heterologous infection-reinfection. We refer to this as a seasonal H1N1 (sH1N1) imprinting with a 2009 H1N1 reinfection (sH1N1->2009 H1N1). Since, influenza B viruses have co-circulated during A virus flux, there is further complexity to human influenza virus preimmunity. Studies are only beginning to uncover the dynamics of infection-reinfection permutations on shaping the human preimmune background.

## 6. Experimental Animal and Human Studies of Preimmunity

We know that extrapolating results from naïve animal influenza virus studies to human disease is limited due the existence of human influenza preimmunity [87]. Below we review the recent literature of sequential influenza virus infection using laboratory-controlled experiments with animal models of influenza preimmunity. The conclusions are then synthesized with findings from human serological and blood cell surveys creating a map of the preimmune host-virus interaction.

Monsubtypic homologous (MsHo) reinfection occurs when a preimmune host is re-exposed to the same or slightly drifted influenza virus strain. To investigate the nuanced humoral antibody changes that are elicited during MsHo reinfection with a drifted virus, Linderman and Hensley exposed BALB/c mice to inactivated A/PuertoRico/8/1934 virus and 28 days later performed a re-exposure with a drifted strain, A/PuertoRico/8/1934-S12a [88]. There are 13 amino acid changes between the two viruses and all mutations were located in the HA head. Since the majority of the epitopes remained constant, this experimental strategy allowed the humoral responses to be analytically parsed in respect to antigenic variability. Both previously existing antibodies, as well as novel antibodies, were elicited during the MsHo re-exposure, suggesting that both the reactivation of memory responses as well as novel immune targeting occur during MsHo reinfection of drifted strains [88].

Using mice, ferrets, and guinea pig models, monosubtypic heterologous (MsHe) infection-reinfection studies have revealed that an infection with the sH1N1 viruses (circulating 1977 to 2009) leads to partial protection upon reinfection with the 2009 pandemic H1N1 “lineage” (2009 H1N1) [13,89,90,91,92,93]. Reduction in disease severity in the sequential sH1N1->2009 H1N1 infections was shown in these studies by minimal weight loss, decreased lung viral load, and decreased lung pathology in mice and ferrets [13]. Fang reported that primary infection induced preexisting non-HA antibodies which were elicited during 2009 H1N1 virus challenge due to sequence homology of the less immunogenic internal viral proteins. Focusing on the HA protein, other reports showed that MsHe sequential infections of the same permutation (sH1N1->2009 H1N1) led to the generation of both HA head and stalk directed antibodies [14,33]. Another report synthesizing human and ferret data also investigated sH1N1->2009 H1N1 infection sequences but focused on the specific sH1N1 epitopes that may invoke a stalk-specific response during re-exposure [94]. This report showed that people imprinted with a historical H1N1 virus containing HA K133 amino acid (a.a.) mounted a dominant antibody response directed toward the HA receptor binding region in the head during secondary exposure [94]. The sH1N1 strains with a K133 a.a. signature circulated from 1983 to 1996. K133 a.a. is also present in many 2009 H1N1 viruses representing a point of cross-reactivity. Conversely, people imprinted with sH1N1 viruses outside of this time period (before 1983 or after 1996) had a response dominated by antibodies directed toward the HA stalk during 2009 H1N1 infection [94]. These findings were confirmed with ferret experimental data recapitulating the human immune backgrounds and analyzing antibody specificity. Together the data from MsHe sequential infection studies suggested that if the reinfection strain has antigenically-divergent epitopes in the HA head, the memory B cells that were previously generated to the stalk will be preferentially recalled over establishing new head specific antibodies.

To gain an appreciation for the MsHo memory responses versus primary immune signatures, gene expression from ferrets exposed to homologous reinfection of 2009 H1N1->2009 H1N1 (21 day recovery period) were compared to the immune responses of ferrets primarily infected with 2009 H1N1 and sH1N1 separately [51]. Global immune responses in ferrets were evaluated by gene expression profiling. MsHo reinfection 21 days following the initial exposure led to decreased interferon and innate immune responses indicating the inhibition of viral infection. Specifically, the chemokine (C-X-C motif) ligand-10 (CXCL10) and chemokine (C-C motif) ligand-5 (CCL5) expression which are typical immune markers of viral load were absent day 1 post infection. Furthermore, there was robust gene induction of the T and B cell associated transcripts IGHM (Ig mu chain C region), IGHG (Ig gamma chain C region), and CD8A during MsHo reinfection, indicating the stimulation of previously generated adaptive immune cells [51]. These signatures can be used as a reference to understand the global responses to specific sequential viral infection combinations in future studies.

In comparison, a recent report by Kosikova and colleagues investigated the antibody dynamics in a MsHo H3N2 virus drift study [95]. Repeated exposure to drifted H3 antigens affected antibody activity and reactivity was investigated in both humans and experimental preimmune animal studies [95]. Human sera were collected from infants (8–30 months of age) and adults (28–74 years of age) following seasonal influenza virus vaccination. The samples were subjected to HAI and microneutralization assays. Experimentally, male ferrets were infected every two weeks (MsHo, H3N2 -> H3N2) starting at approximately four months of age. This age represents neither youth nor adulthood for ferrets since ferrets are considered infants prior to 8 weeks and adults after six months of age [96]. The evolving immune responses to drifting H3N2 virus was analyzed by comparing antibody reactivity following infection or vaccination with viruses of the currently drifting H3N2 clades 3C.2a, 3C.3a, 3c.1, and 1. The researchers found that sequential homologous H3N2 strain infections led to epitope narrowing of the elicited antibodies which complemented their human serology results. Together, these data suggest that preimmunity acquired by frequent exposure to H3N2 drifting viruses by homologous re-exposure leads to less broadly reactive antibodies. Taken together with the H1N1 MsHo drift study [88], the results suggest that humoral immune responses may differ according to the drifting subtype.

**Figure 4:**
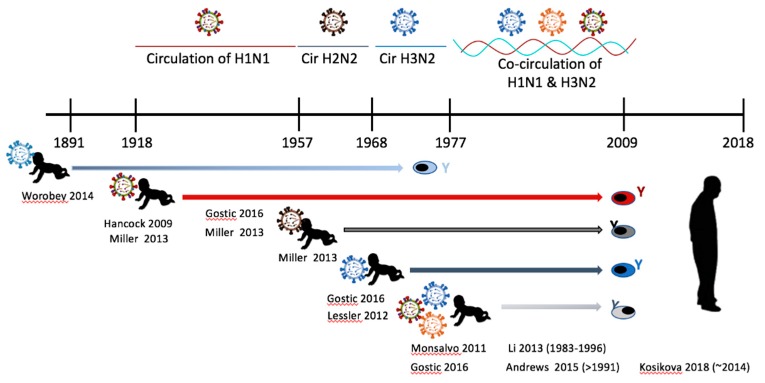
Influenza immune imprinting and the history of circulating influenza viruses. The timeline illustrates the relationship between virus circulation and influenza virus immune imprinting at infancy. Several published studies have shown that antibodies reactive toward the imprinting virus can last throughout the host’s lifetime. Human studies investigating imprinting serology, epidemiology, or cell reactivity are noted in the figure for the time period of imprinting. The author’s last name and publication date are given. The date in parentheses refers to study subjects’ date of birth if it is not easily deduced from the timeline.

Heterosubtypic sequential infections (example H1N1 -> H5N1) have also been shown to induce antibody responses directed toward the HA stalk [16]. Epidemiological data has shown that people imprinted with HA group 1 H1N1 viruses prior to 1957 were protected from severe disease by later infection with high pathogenic avian viruses (H5N1) (Figure 4). Since H5N1 is also a group 1 virus, the authors hypothesize that the order of virus infection H1N1->H5N1 will invoke a strong antibody response directed toward the HA stalk since the head region is too divergent to recall HA head reactive memory B cells. The authors also gave evidence that those born during the circulation of H3N2, a group 2 HA virus, would not be protected from H5N1 [16]. Together the authors concluded that activation of stalk-specific memory B cells was the immunological mechanism leading to protection from highly pathogenic H5N1 infection. This study and others involving human serology and epidemiology are placed in historical context in Figure 4. Another study identified a human monoclonal antibody that was extremely broadly reactive toward the stem of both group1 and group2 viruses [97]. This antibody (MED1882) was found to use the heavy chain VH6-1 gene and was functionally protective during adoptive transfer experiments in mice and ferrets. The antibody binds to the virus in the hydrophobic groove of the HA protein fusion domain. The authors hypothesize that the generation of this antibody occurred following sequential influenza infections which occurred during the donor’s lifetime which encompassed exposures to sequential MsHe viruses in the course of historical influenza pandemics: H2N2 -> H3N2 -> H1N1. Relatedly, other researchers have leveraged the sequential infection strategy for generating stalk specific antibody responses [31,98,99]. By generating stalk reactive antibodies, a wider breadth of protection against evolving influenza viruses may be achieved [31,99]. Specifically, one strategy has been to prime the host with HA molecules from one virus strain and boost with a chimeric HA molecule containing the stalk region from the original influenza HA protein but an HA head containing little a.a. homology (exotic head) [99]. This exposure order will predominantly stimulate memory B cells producing antibodies targeted to the stalk representing a possible vaccination strategy.

Although the human immune background is far more complex than that of experimental animal models, personal influenza virus exposure history can be deduced. As discussed previously, cross-referencing a person’s birth year against the history of influenza A and B virus circulation we can surmise a personal preimmune complexion [10,12,14,16,18,75,94]. A study analyzed human sera antibody reactivity following vaccination or infection by computationally comparing antibody reactivity against the antigenic a.a. similarity among viruses to create a visual antibody landscape [8]. Following analysis, the authors concluded that reactivity of previously established B cell clones is dependent on the order of virus type and subtype exposures [8]. Another study reported a role for non-protective antibodies in immune complexes (IC)-mediated disease following pandemic 2009 H1N1 infection in humans [100]. Severely ill patients infected with the 2009 H1N1 virus had high titers of low avidity serum antibodies which were previously generated from sH1N1 exposures. This study found that the fatal cases were marked by IC in the respiratory tract. The data suggested that antibodies previously generated from past seasonal influenza virus infections (MsHe infection) had a negative impact on disease progression during 2009 H1N1 infection. Looking at the B cell biology and source of the antibody responses, another study investigated the cell type responsible for antibody generation during 2009 H1N1 virus vaccination in humans [14]. There were two major findings of this study. The first was that people with low initial 2009 H1N1 antibody titers generated a broadly reactive antibody response directed at the HA stalk, whereas those with high 2009 H1N1 antibody pre-vaccination titers with a head directed response following vaccination. The stalk antibodies were polyreactive and their magnitude decreased during repeated 2009 H1N1 vaccination. The authors hypothesized these antibodies became less abundant due to the decreased accessibility of the HA stalk region which would allow for less cell stimulation leading to anergy of the antibody producing B cell clone. The second major finding was that the precursor for short-lived plasmablasts were memory B cells [14]. In summary, these human studies suggest that the elicitation of antibodies and their specificity during a secondary or tertiary exposure is dependent on the specific antigenic variation of each virus in sequence. 

Each year, H1N1, H3N2, B-Yamagata, and B-Victoria influenza virus strains circulate in the human population. Despite the continuing circulation of both A and B influenza viruses, there is a paucity of information regarding heterotypic infection-reinfection events. This is surprising since sequential heterotypic infections in the human population are possible both interseasonal as well as intraseasonal. In the northern hemisphere, the influenza season begins in October lasting until April and peaks early January. Typically, in each season influenza A cases are prominent in the first half to three quarters of the season [101,102]. As influenza A human cases begin to decline, there is an increase in the prominence of influenza B viruses [101,102] suggesting viral interference or the opportunistic infection of influenza B viruses in immunosuppressed hosts. One human study documented sequential infections in the same host. Specifically, children who required medical intervention were documented to have been infected with an influenza A strain and then reinfected with an influenza B virus during the same season [103]. All children in this retrospective study were immunocompetent and the interval between infections was ~50 days. This study provides evidence that the heterotypic infection-reinfection occurs at an observable frequency and can lead to severe disease without cross-protection. Moreover, other studies have showed prominent co-infections of influenza A and influenza B viruses [104]. Further study is needed to understand the mechanisms of immune activation or suppression during sequential heterotypic and co-infections of two influenza virus types.

## 7. Vaccination Preimmunity May Influence Future Vaccination Outcome

Despite immune history, influenza viruses are a recurrent and as yet unsolved public health problem. These viruses cause millions of hospitalizations and thousands of deaths each year estimated at 500,000 people worldwide by the WHO [79,102,105]. Yearly influenza vaccination is recommended by the National Advisory Committee on Immunization (NACI) of Canada, the Advisory Committee on Immunization Practices (ACIP) in the United States, and the World Health Organization (WHO) as the most effective strategy for reducing influenza burden [22,106,107]. The low effectiveness of the seasonal influenza vaccine has been attributed to the frequent mutations in the virus which occurs through antigenic drift [108]. Aside from viral antigenic changes causing low vaccine effectiveness (VE), low VE has previously been linked by some researchers to repeated seasonal influenza vaccination in specific antigenically-defined influenza seasons [11]. These effects of repeated vaccination was first publicized in 1979. Data from a study following schoolboys found that subjects who were sequentially vaccinated for H3N2 virus had no statistically determined protective advantage compared to unvaccinated children during seasonal influenza virus circulation [9,109]. Loss of protection due to repeated vaccination has been named the Hoskins Paradox referring to the lead author of the 1979 study. More recently, VE following sequential seasonal vaccination has been investigated in two independent reports [11,110]. These reports use the Antigen Distance Hypothesis (ADH) as the metric to determine the influence of preimmunity acquired through vaccination in previous years against the current vaccine. The influence of vaccine-established preimmunity is determined by comparing the antigenic relatedness between the reference viruses of previous and current vaccines. ADH suggests that if there is minimal antigen relatedness between previous and current vaccination then there will be increased antigen interference from previous memory B cell clones. Conversely, increased antigenic diversity will permit the generation of new B cell clones and positive outcome for vaccine protection. In both reports the authors showed epidemiological evidence that repeated vaccination during seasons with antigenically-related vaccine reference strains led to a greater percentage of vaccinated people testing positive for influenza virus infections [11]. The evidence supporting the antigen distance hypothesis diverges from the findings of Monsalvo and colleagues [100] who showed that previously existing low avidity antibodies that were elicited from large antigenic changes were deleterious during new antigenically-divergent 2009 H1N1 virus infections. It is possible that this discrepancy is due to differential immune mechanisms of antigenic distance initiated between vaccination and live virus infection. The antigenic distance hypothesis as a mechanism of reduced effectiveness for specific influenza seasons remains a hypothesis and is not presented here as leverage for skipping influenza virus vaccinations. More studies of vaccination effectiveness in other countries, as well as experimental studies directly testing the antigenic distance hypothesis is needed. Furthermore, there are numerous publications supporting the regular use of influenza vaccinations in children, adults, and pregnant women following rigorous scientific study. Results from these analyses support vaccine induced protection against severe disease following influenza virus infection, as well as improved neonatal outcomes when vaccination occurred during pregnancy [111,112,113,114].

## 8. Future Directions

Influenza viruses are an ever-constant threat throughout a host’s lifetime. Reviewing the literature shows that there are several knowledge gaps that could be addressed in future studies. Specifically, there is a lack of experimental studies in three areas: (1) memory phase exposures, (2) imprinting in infancy versus adulthood, and (3) immune memory mechanisms of subsequent infections.

Previous studies investigating the immune re-encounter with influenza viral antigens or live virus infection are frequently designed with short recoveries of 14 to 28 days separating primary and secondary challenges [88,95,115]. These time frames do not properly account for the actual immunological recovery. Although these studies offer insight into secondary immune responses, the interval of time between sequential infections or vaccinations does not accurately reflect the human immune condition of yearly viral challenge [70]. Enough recovery time (more than two months in animal studies) should be given to allow for nonspecific immune responses to subside. At this point only a small set of specific memory cells would be present at the time of secondary pathogen exposure. This design would more accurately reflect the seasonal exposures of influenza viruses in humans. Longer recovery times for animal experiments are costly in time and money which may be why they are rarely approached. We suggest that more studies examining re-exposure during the adaptive immune memory phase will uncover immune mechanisms relating influenza pathogenesis. Furthermore, since most studies are focused on the viral HA and the humoral responses targeting this protein, it will also be important that future studies give more attention to the alternative immune responses. Specific adaptive immune components of interest are CD8^+^ T cells, CD4^+^ Th1 cells, and memory NK cells [68]. The major hurdle to understanding the responses of these alternative adaptive memory cells is the lack of immune correlates for their responses and functions. The standard methodology for assessing immune activation following influenza virus infection is the HAI (hemagglutinin inhibition) assay which is associated with B cell activity. Furthermore, this assay only determines the minimum concentration of serum antibodies required to inhibit a virus of interest from binding to sialic acids present on red blood cells [116]. The assay does not evaluate all of the possible viral targets of the humoral antibody responses or antibody functions, such as virus neutralization [116]. Basic discovery studies are needed to identify correlates of NK memory cell, CD8+ T cell, non-HA antibody reactivity, and protection.

Immune regulation in infants and children is significantly skewed toward Th2 responses and highly regulated by the interleukin-10 (IL-10) and transforming growth factor β (TGF-β) response network compared to adults [117,118,119]. Furthermore, it is well known that children and infants have different clinical outcomes to influenza virus infection and vaccination compared to adults [120,121,122]. Infants and young children are high-risk groups for developing severe respiratory disease leading to hospitalization following influenza virus infection [102,107], which may be due to the immature respiratory tract or immune system in the young. Tissue damage in the lung can obstruct the narrow immature airway, cause surfactant deficiency, and lead to collapsed lungs and respiratory distress [120]. Influenza imprinting occurs during early childhood, not during adulthood in humans [16,75]. Looking at the experimental studies, we see that the majority of studies using in vivo models utilize animals of adult age. Limited studies by us and others [123,124] have investigated the immune responses of younger animals and shown age-specific immune responses such as the development of iBALT (inducible Bronchus Associated Lymphoid Tissue) in the lungs of young animals following influenza virus infection [125,126]. Despite this evidence, we are unaware of any sequential influenza animal models that imprint during infancy and reinfect during adulthood. The respiratory tract of infants is significantly different from that of the adult, and responds differently to insult. Since the infant lung is not considered mature until 36 months postpartum [127], it is essential that animal models of recurrent influenza infection take age into account. Having a more complete understanding of influenza infection during youth and the immunity gained will guide vaccine development.

Our understanding of the mechanisms of immune memory for antigenically variable and dynamic pathogens such as influenza viruses is limited. The human studies from Andrews and colleagues have given evidence that the precursor to plasmablasts initiated by vaccination is the memory B cell [14]. This leaves several questions regarding the mechanisms of memory B cell development as well as activation and initiation of plasmablast function. Not all memory B cells are the same. Some are resident within the primary infected tissue while others circulate in the periphery [65,128,129]. Furthermore, memory B cells may be germinal center dependent or independent. It would be insightful to determine if antigenic relatedness across sequential virus infections influences memory B cell pathways, development, and recall. Moreover, what are the signaling pathways leading to memory B cell transformation into plasmablasts? Is activation dependent on the antigenic variation in the viral exposure sequence? Researching these questions may uncover molecular key drivers of this immune refinement process. Identification of key drivers of immune refinement for dynamic pathogens may suggest targets for improved vaccine development.

## 9. Concluding Statement

The host pathogen interaction of antigenically-evolving and variable influenza viruses leads to a complex host immune background. Preimmunity blends age, immune function, and sequence of influenza virus infections. By analyzing consecutive influenza virus exposures as monosubtypic homologous, monosubtypic heterologous, heterosubtypic, or heterotypic sequential infections we may identify immune mechanistic patterns that govern future responses. Ultimately, we want to study preimmunity and understand the mechanisms to guide future vaccine design and offer insight into an effective universal influenza vaccine. Although this has been the strategy of universal influenza vaccine development targeting the HA stalk domain [61], there are still many knowledge gaps regarding the mechanisms of immune imprinting and preimmunity. We have highlighted three areas of investigation: memory phase recovery, imprinting age, and memory B cell mechanisms. Findings from animal models of sequential influenza virus infection may be extrapolated to the knowledge gaps of human studies. Understanding how the human immune response changes over sequential influenza infections and vaccinations will inform the rational development of more efficacious vaccines and vaccination policies for the public.

## Figures and Tables

**Table 1:** Influenza virus relationships of sequential infection.

Infection Relationship	Example Sequence	Example Strain 1	Example Strain 2
**Monosubtypic Homologous**	sH1N1 -> sH1N1	A/Brisbane/59/2007	A/Brisbane/59/2007
**Monosubtypic Heterologous**	sH1N1 -> 2009 H1N1	A/Brisbane/59/2007	A/California/07/2009
**Heterosubtypic**	sH1N1 -> H3N2	A/FortMonmouth/1/1947	A/HongKong/1/1968
**Heterotypic**	sH1N1 -> B-V virus	A/Brisbane/59/2007	B/Brisbane/60/2008
